# Potential Involvement of Snail Members in Neuronal Survival and Astrocytic Migration during the Gecko Spinal Cord Regeneration

**DOI:** 10.3389/fncel.2017.00113

**Published:** 2017-04-24

**Authors:** Tingting Shen, Yingjie Wang, Qing Zhang, Xue Bai, Sumei Wei, Xuejie Zhang, Wenjuan Wang, Ying Yuan, Yan Liu, Mei Liu, Xiaosong Gu, Yongjun Wang

**Affiliations:** Key Laboratory of Neuroregeneration of Jiangsu and Ministry of Education, Co-innovation Center of Neuroregeneration, Nantong UniversityNantong, China

**Keywords:** snail, spinal cord, regeneration, neuron, glial cells

## Abstract

Certain regenerative vertebrates such as fish, amphibians and reptiles are capable of regenerating spinal cord after injury. Most neurons of spinal cord will survive from the injury and regrow axons to repair circuits with an absence of glial scar formation. However, the underlying mechanisms of neuronal anti-apoptosis and glia-related responses have not been fully clarified during the regenerative process. Gecko has becoming an inspiring model to address spinal cord regeneration in amniotes. In the present study, we investigated the regulatory roles of Snail family members, the important transcriptional factors involved in both triggering of the cell migration and cell survival, during the spontaneous spinal cord regeneration. Both Snail1 and Snail3 have been shown to promote neuronal survival and astrocytic migration *via* anti-apoptotic and GTPases signaling following gecko tail amputation. Transforming growth factor-beta (TGFβ), together with other cytokines were involved in inducing expression of Snail protein. Our data indicate a conserved function of Snail proteins in embryonic development and tissue regeneration, which may provide clues for CNS repair in the mammals.

## Introduction

Unlike mammals, the regenerative model organisms including fish, amphibian and several reptiles are capable of regenerating spinal cord throughout their lifespan after injury (Dong et al., 2013; Lee-Liu et al., 2013; Szarek et al., 2016; Rasmussen and Sagasti, 2017). Spinal cord injury (SCI) by transection, resection, compression or tail amputation in these animals, will results in a robust ability to regrow axons, repair circuits and recover function (Díaz-Quiroz and Echeverri, 2013). Either surviving or newly generated neurons grow axons along the channels present in regenerating ependymal glial cells, in a permissive milieu with limited myelin-derived inhibitory factors, and a lack of glial scar formed by reactive astrocytes (Egar and Singer, 1972; Singer et al., 1979; Ferretti et al., 2003; Popovich and Longbrake, 2008; Díaz-Quiroz and Echeverri, 2013; Rasmussen and Sagasti, 2017). It has been generally regarded that the “real” stellate astrocytes are absent from anamniotes (Lyons and Talbot, 2014), in spite of some unrepeatable adverse reports (Kawai et al., 2001; Alunni et al., 2005). Works in *Gekko japonicus*, a reptile, have revealed that the animal can regenerate spinal cord following injury, without evoking astrocytic responses (Gao et al., 2010; Gu et al., 2015). The molecular cues with respect to promoting the neuronal survival and mediating the events of non-reactive astrocytes, have not been fully clarified in the regenerative animals.

Development-related genes have been found to regulate appendage regeneration, strengthening the postulation that epimorphic regeneration recapitulates development (Stocum, 1984; Beck et al., 2003; Lozito and Tuan, 2015, 2016; Alibardi, 2016, 2017). Snail proteins constitute an evolutionarily conserved superfamily of zinc-finger transcription factors including snail1, snail2, snail3 and Scratch (Kerner et al., 2009). Structurally, proteins of this family share a high degree of homology at the C-terminal region, containing four to six C2H2 zinc fingers, and at the N-terminal region that contains the SNAG transactivation domain. This conserved SNAG domain extends to the nine amino acids, which is essential for their nuclear localization and for Gfi1-mediated transcriptional repression (Grimes et al., 1996; Manzanares et al., 2001). Snail2 exclusively contains a specific 28 amino-acid sequence called the SLUG domain (SLUG) with unknown function (Manzanares et al., 2001). Snail members play key roles during embryonic development, besides their best known function in carcinogenesis and metastasis by triggering of epithelial-mesenchymal transition (EMT; Gupta et al., 2005; Kurrey et al., 2005; Pérez-Mancera et al., 2005). These transcriptional factors have been found to affect development in the migration of neural crest cells, in the control of mesoderm specification, in the EMT of mesodermal cells, and in the rescue of hematopoietic cells from apoptosis (Le Douarin et al., 1994; Nieto et al., 1994; Mayor et al., 1995; Inoue et al., 2002; Nieto, 2002; Barrallo-Gimeno and Nieto, 2005). Also, the proteins are able to coordinately regulate the survival, self-renewal and differentiation of radial glial precursor cells in the embryonic murine cortex (Zander et al., 2014). Whether Snail proteins contribute to the spinal cord regeneration remains elusive.

Reptiles are located at a significant evolutionary position bridging lower vertebrates and mammals. Similar to fishes and amphibians, several adult species in this lower amniotes are able to regenerate spinal cord, characterized by long neural tracts, nerve cells, supportive glial cells and a central canal with surrounding stem cells (Ferretti et al., 2003; McLean and Vickaryous, 2011). In the present study, we used *Gekko japonicus* as an experimental SCI model to investigate the involvement of Snail family members in regulation of spinal cord regeneration. Our results demonstrated both Snail1 and Snail3 were activated by cytokines including transforming growth factor-beta (TGFβ) following SCI, which in turn play roles of apoptotic repression in neurons and migratory enhancement in glial cells, thus might promote spontaneous spinal cord regeneration.

## Materials and Methods

### Animals

Adult *Gekko japonicus* were used as described by Dong et al. (2013). Briefly, adult animals were fed *ad libitum* with mealworms and housed in an air-conditioned room with a controlled temperature (22–25°C) and saturated humidity. Anesthesia was induced by cooling the animals on ice prior to tail amputation. Amputation was performed at the sixth caudal vertebra, identified based on the special tissue structure present at that position (McLean and Vickaryous, 2011), by placing a slipknot of nylon thread and pulling gently until the tail was detached, thus mimicking the autotomy undergoing for natural defense.

All experiments were conducted in accordance with guidelines of the NIH (Guide for the Care and Use of Laboratory Animal: 1985), and the Guidelines for the Use of Animals in Neuroscience Research by the Society for Neuroscience. Experiments were approved according to the Animal Care and Use Committee of Nantong University and the Jiangsu Province Animal Care Ethics Committee. All geckos (*n* = 15) were anesthetized on ice prior to sacrifice.

### Cloning and Analysis of Snail Family Members

To obtain the full length of gecko Snail family members, anti-sense primer for Snail1 (5′-CAT GCG GGA GAA AGT CCG GGA GCA GGT T-3′), Snail2 (5′- TGT TTG TGC AGA AGA GAC ATG CGG GAG A -3′) and Snail3 (5′- GCA CAT CCG CAC CCA CAC GCT GC -3′) ; sense primer for Snail1 (5′- GAA GCC CAA CTA CAG CGA GCT GGA GAG -3′), Snail2 (5′- ACT TCA AGG ACA CAT CAG AAC TCA CAC C -3′) and Snail3 (5′- TCA AGA TGC ACA TCC GCA CCC ACA CGC T -3′) were designed according to genome sequences (Liu et al., 2015). Both 5′-RACE and 3′-RACE were performed using the BD SMART RACE cDNA Amplification Kit (Clontech, Mountain View, CA, USA) according to the manufacturer’s instructions. Comparison against the GenBank protein database was performed using the PSI-BLAST network server at the National Center for Biotechnology Information (Altschul et al., 1997). Multiple protein sequences were aligned using the MegAlign program by the CLUSTAL method in the DNASTAR software package (Burland, 2000).

### Production of Snail Overexpression Lentivirus

Snail overexpression (LV5-Snail) lentivirus was produced in Shanghai GenePharma Co. Ltd, according to the manufacturer’s procedures. The ORF of snail family members was cloned to the LV5 vector via the Not I and Bam HI sites, respectively. Snail expression was driven by the EF-1α promoter, and the expression of reporter enhanced green fluorescent protein (eGFP) was driven by CMV promoter. Both Snail and eGFP sequence were incorporated into a lentivirus. Lentiviruses were produced using 293T cells, and the viral titers reached 1 × 10^9^ TU/ml for further studies.

### Quantitative Real-Time Polymerase Chain Reaction (Q-PCR)

Total RNA was prepared with Trizol (Gibco, Gran Island, NY, USA) from different tissues, including the brain, spinal cord, heart, liver, testis and ovary of adult geckos. Total RNAs were also extracted from 0.5 cm spinal cord segments of 20 geckos amputated from the sixth caudal vertebra at 1 day, 3 days, 1 week and 2 weeks, respectively.

For Q-PCR examination of *Snail* temporal expression, the first-strand cDNA was synthesized using Omniscript Reverse Transcription Kit (QIAGEN) in a 20 μl reaction system containing 2 μg total RNA, 0.2 U/μl M-MLV reverse transcriptase, 0.5 mM dNTP mix, 1 μM Oligo-dT primer. The cDNA was diluted 1:5 before use in Q-PCR assays. The sequence-specific primers were designed and synthesized by Invitrogen (Shanghai, China). Primer pair and probe for Snail1: forward primer 5′- CCG AGA AAT TCC ACT GCA -3′, reverse primer 5′- GGT ATG GCT TCG GAT GTG -3′; for Snail2, forward primer 5′- TAC CTT TAT GAG AGC TAC CCA -3′, reverse primer 5′- TTC CCA AAG ACG AAG GAT ATC -3′; for Snail3, forward primer 5′- TTA GTT GCT CCG TCC AGA -3′, reverse primer 5′- AAA CCA CGT TGC CAT ACA -3′. Q-PCR reactions were performed in a final volume of 20 μl (1 μl cDNA template and 19 μl Q-PCR reaction buffer containing 2.5 mmol/L MgCl_2_, 0.2 mmol/L dNTPs, anti-sense and sense primers 0.5 μmol/L, taqman probe 0.4 μmol/L, DNA polymerase 0.2 μl and 1 × DNA polymerase buffer). The Rotor-Gene 5 software (Corbett Research, Rotor-Gene, Australia) was used for real-time PCR analysis. Reactions were processed using one initial denaturation cycle at 94°C for 5 min followed by 40 cycles of 94°C for 30 s, 60°C for 30 s and 72°C for 30 s. Fluorescence was recorded during each annealing step. At the end of each PCR run, data were automatically analyzed by the system and amplification plots obtained. Snail full-length plasmid was used to prepare standard curves and used as a specificity control for real-time PCR. The expression levels of the Snail cDNA were normalized to an endogenous EF-1α cDNA using forward primer 5′-CCT TCA AAT ATG CCT GGG T-3′, reverse primer 5′-CAG CAC AGT CAG CTT GAG AG-3′ and taqman probe 5′-TTG GAC AAG CTG AAG GCA GAA CGT G-3′. In addition, a negative control without the first-strand cDNA was also performed.

### Cells Culture and Treatment

Human neuroblastoma cell line SH-SY5Y (Chinese Academy of Sciences, Shanghai Institutes for Biological Sciences Cell Resource Center) or gecko astrocytes cell line, Gsn1, were grown in Dulbecco’s Modified Eagles Medium (DMEM, Gibco BRL) supplemented with 10% (v/v) fetal bovine serum in a 37°C or 30°C humidified incubator with 5% CO_2_. For glucose deprivation (GD)-induced neuronal apoptosis, differentiated SH-SY5Y cells induced with 1 μM all trans-retinoic acid (RA, Sigma) were transfected with LV5-Snail or control lentivirus, and cultured in DMEM with medium supplemented with 5% (v/v) fetal bovine serum. Then, they were subjected to GD insult as described previously (Ferretti et al., 2016). Cells were transferred to the glucose-free DMEM in the incubator chamber for 24 h incubation. At the end of cell treatments, cell culture was subjected to various assessments, or counterstained with annexinV-PE for 10 min at 20–25°C and mounted on slide glasses with mounting medium. Images were captured on a Nikon Diaphot microscope.

Gsn1 cells were treated with 4 ng/ml recombinant TGFβ1 or TGFβ2 (Peprotech) with or without 10 μmol/L TGFβ receptor inhibitor LY2109761 (Selleck) for 2 h, respectively. The cells were then subjected to the determination of Snail transcriptional expression.

### Cell Proliferation Assay

Gsn1 cells were resuspended in fresh pre-warmed (30°C) complete medium, counted and plated at a density of 2 × 10^5^ cells/ml on 0.01% poly-L-lysine-coated 96-well plates. At the indicated time point after cell transfection, 50 mM EdU was applied to the cultures and the cells were grown for an additional 2 h. Finally, the cells were fixed with 4% formaldehyde in PBS for 30 min. After labeling, the Gsn1 cells were assayed using Cell-Light EdU DNA Cell Proliferation Kit (Ribobio) according to the manufacturer’s protocol. Analysis of Gsn1 proliferation (ratio of EdU^+^ to all Gsn1 cells) was performed using images of randomly selected fields obtained on a DMR fluorescence microscope (Leica Microsystems, Bensheim, Germany). Assays were performed three times using triplicate wells.

### Cell Migration Assay

Migration of astrocytes was studied using 6.5 mm transwell chambers with 8 μm pores (Corning Costar) as described previously (Dong et al., 2013). One hundred microliters astrocytes (2 × 10^5^ cells/ml) resuspended in DMEM/F12 were transferred to the top chambers of each transwell and allowed to migrate at 30°C in 5% CO_2_ for 30 h, and 600 μl of DMEM/F12 was injected into the lower chambers. The upper surface of each membrane was cleaned with a cotton swab at the indicated time point. Cells adhering to the bottom surface of each membrane were stained with 0.1% crystal violet, imaged, and counted using a DMR inverted microscope (Leica Microsystems). Assays were done three times using triplicate wells.

### Western Blot

Protein was extracted from cells with a buffer containing 1% SDS, 100 mM Tris–HCl, 1 mM PMSF and 0.1 mM β-mercaptoethanol. Protein concentration of each specimen was detected by the Bradford method to maintain the same loads. Protein extracts were heat denatured at 95°C for 5 min, electrophoretically separated on 10% SDS–PAGE, and transferred to PVDF membranes. The membranes were subjected to the reaction with a 1:1000 dilution of primary antibodies in TBS buffer at 4°C overnight, followed by a reaction with secondary antibody conjugated with goat anti-rabbit or goat anti-mouse HRP (Proteintech) dilution 1:1000 at room temperature for 2 h. After the membrane was washed, the HRP activity was detected using an ECL kit. The image was scanned with a GS800 Densitometer Scanner (Bio-Rad), and the data were analyzed using PDQuest 7.2.0 software (Bio-Rad). β-actin (1:5000) was used as an internal control. Antibodies used in Western blot are: Snail1 (polyclonal antibody prepared from polypeptides), Snail3 (polyclonal antibody prepared from polypeptides); GFAP (Sigma); Cleaved caspase 3 (Asp175), *p*-ERK1/2, ERK1/2, *p*-AKT, AKT, Bcl-XL (Cell Signaling), N-cadherin and β-actin (Proteintech).

RhoA, Rac1 or Cdc42 activation was determined using the rhotekin-RBD that specifically binds activated Rho and the PBD-PAK that has a high affinity for both GTP-Rac and GTP-Cdc42 (RhoA/Rac1/Cdc42 Activation Assay Combo Biochem Kit, Cytoskeleton, Denver, CO, USA). In brief, the cells were lysed with ice-cold cell lysis buffer containing 50 mM Tris-HCl, pH 7.4, 2 mM MgCl_2_, 1% NP-40, 10% glycerol, 100 mM NaCl, and Protease Inhibitor Cocktail (Roche Diagnostics, Basel, Switzerland) and centrifuged for 5 min at 14,000 g. The equivalent protein amounts of lysate (500 μg total cell protein) were performed pull-down assay with 50 μg rhotekin-RBD beads and 20 μg PAK-PBD beads, and rotated for 60 min at 4°C. The beads were washed three times with lysis buffer and heated for 5 min at 100°C in SDS-PAGE sample buffer, and then analyzed for bound RhoA, Rac1 and Cdc42 molecules by Western blotting using anti-RhoA antibody (1:500, Cytoskeleton), anti-Rac1 antibody (1:500, Cytoskeleton) or anti-Cdc42 antibody (1:500, Cytoskeleton).

### Tissue Immunohistochemistry

The spinal cord segments were harvested, post-fixed and sectioned. Sections were allowed to incubate with ployclonal rabbit anti-gecko Snail1, rabbit anti-gecko Snail3 antibody (1:500 dilution), polyclonal rabbit anti-bovine galactocerebroside antibody (1:200 dilution, Millipore), polyclonal rabbit anti-human GFAP antibody (1:500 dilution, Sigma), or polyclonal rabbit anti-human neuron-specific enolase (NSE) antibody (1:200 dilution, Abcam) at 4°C for 36 h. The sections were further reacted with the Cy3-labeled secondary antibody goat anti-mouse IgG (1:400 dilution, Gibco), or the FITC-labeled secondary antibody goat anti-rabbit IgG (1:400 dilution, Gibco) at 4°C overnight, followed by observation under a confocal laser scanning microscope (Leica, Heidelberg, Germany).

### Statistical Analysis

Statistical significance of differences between groups was analyzed by one-way analysis of variance (ANOVA) followed by Bonferroni’s *post hoc* comparisons tests with SPSS 15.0 (SPSS, Chicago, IL, USA). Normality and homoscedasticity of the data were verified before any statistical analysis using levene’s test. Statistical significance was set at *p* < 0.05 level (significant) and *p* < 0.01 (highly significant).

## Results

### Three Snail Paralogs are Recovered in Gecko

**Figure 1:**
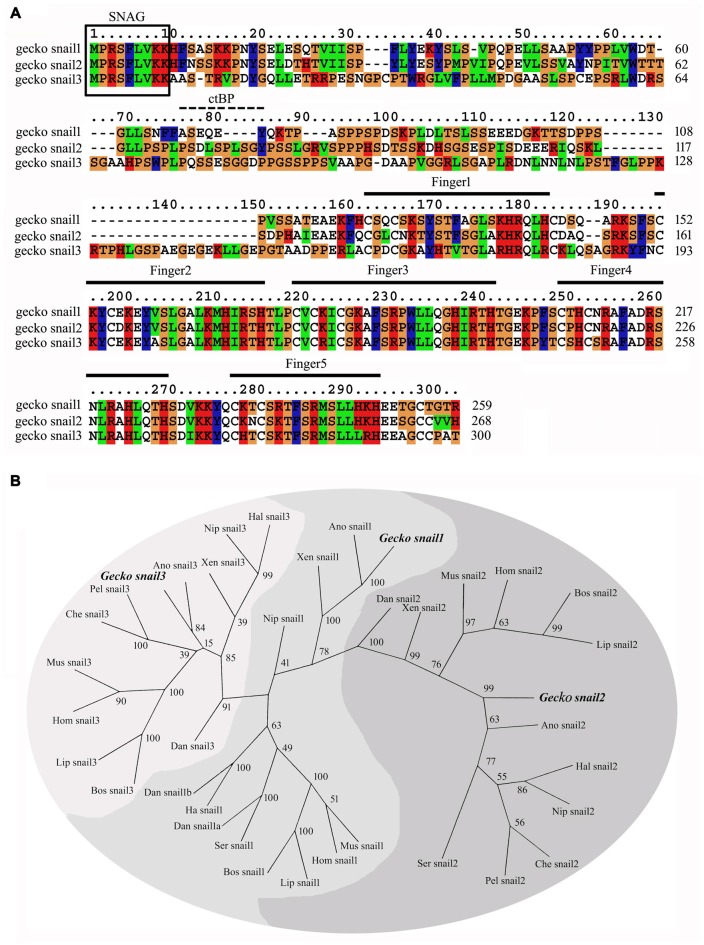
**Sequence analysis of the gecko Snail paralogs. (A)** Alignment of the deduced amino acid sequence of gecko Snail1, Snail2 and Snail3. Gaps introduced into sequences to optimize alignment are represented by dashes. The zinc-finger domains are indicated in the figure from the first cystidine of the finger domain to the last histidine. The conserved SNAG domain is boxed. The CtBP interaction motif is indicated with dashes; **(B)** unrooted phylogenetic tree of gecko Snail and those of other known Snail proteins from representative species constructed by the neighbor-joining method within the package PHYLIP 3.5c. Bootstrap majority consensus values on 1000 replicates are indicated at each branch point in percent. Sequences obtained from GenBank or Swissprot are gecko *Gekko japonicus* Snail1 (KT032183), Snail2 (KT032184), Snail3 (KT032185); human *Homo sapiens* Snail1 (NP_005976), Snail2 (NP_003059), Snail3 (NP_840101); mouse *Mus musculus* Snail1 (NP_035557), Snail2 (NP_035545), Snail3 (NP_038942); cattle *Bos taurus* Snail1 (NP_001106179), Snail2 (NP_001029710), Snail3 (NP_001179562); dolphin *Lipotes vexillifer* Snail1 (XP_007446447), Snail2 (XP_007464078), Snail3 (XP_007468498); bird *Nipponia nippon* Snail1 (XP_009463837), Snail2 (XP_009471359), Snail3 (XP_009475765); bird *Haliaeetus* *leucocephalus* Snail1 (XP_010567958), Snail2 (XP_010572691), Snail3 (XP_010578013); bird *Serinus canaria* Snail1 (XP_009091543), Snail2 (XP_009101147); green anole *Anolis carolinensis* Snail1 (XP_003220701), Snail2 (XP_003223554), Snail3 (XP_003228606); turtle *Pelodiscus sinensis* Snail2 (XP_006112231), Snail3 (XP_006125863); turtle *Chelonia mydas* Snail2 (XP_007058841), Snail3 (XP_007059405); frog *Xenopus tropicalis* Snail1 (NP_989267), Snail2 (NP_989424), Snail3 (XP_002933720); zebrafish *Danio rerio* Snail1a (NP_571141), Snail1b (NP_571064), Snail2 (NP_001008581), Snail3 (NP_001070853).

By screening genome of gecko, three Snail paralogs, namely Snail1, Snail2 and Snail3, are recovered. The full sequence of Snail1 (GenBank accession number **KT032183**), Snail2 (**KT032184**) and Snail3 (**KT032185**) amplified by 5′- and 3′- RACE, encodes a protein of 259, 268 and 300 amino acid residues, respectively (Figure 1A). All these paralogs contain the N-terminal SNAG domain, a short sequence of nine amino acids (amino acids 1–9) associating with transcriptional repression (Manzanares et al., 2001). The CtBP (C-terminal Binding Protein) interaction motif, which is prevalent in invertebrates to facilitate interaction with the co-repressor C-terminal Binding Protein (Hemavathy et al., 2000), was also present in gecko Snail2 and Snail3 (Figure 1A), in concert with the findings from *Xenopus*, chicken, mouse and human Snail2 (Hemavathy et al., 2000). Gecko Snail paralogs possess five C2H2 zinc finger motifs at the C-terminal domain (Figure 1A), while only four such motifs are found in human Snail1, suggesting differential binding affinities of gecko Snail1 to target genes (Sefton et al., 1998; Villarejo et al., 2014).

Phylogenetic tree constructed using the PHYML implementation of Maximum-Likelihood demonstrated that gecko Snail paralogs clustered with corresponding homologs of other vertebrates, suggesting an evolutionary conservation of the protein in the phylogeny (Figure 1B).

### Expression Analysis of Snail Paralogs in the Regenerating Spinal Cord

**Figure 2:**
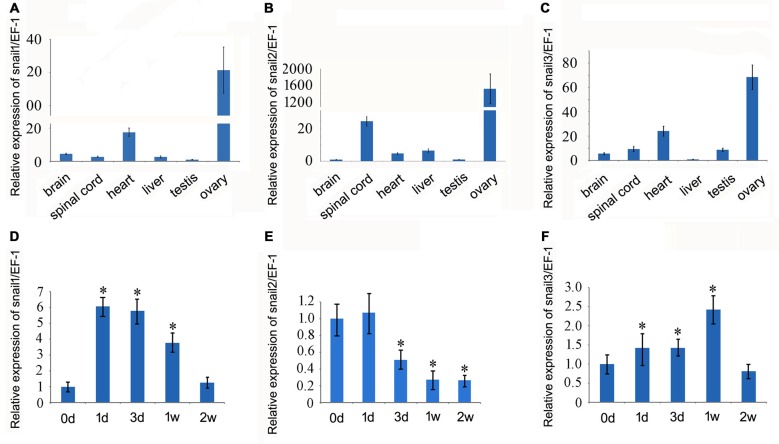
**Real-time PCR analysis of *Snail* transcripts in different gecko tissues and in the regenerating spinal cord. (A–C)** *Snail* expression in different gecko tissues; **(D–F)** *Snail* expression in the spinal cord following tail amputation at 0 day, 1 day, 3 days, 1 week and 2 weeks. Data are expressed as mean ± SEM; *p < 0.01.

**Figure 3:**
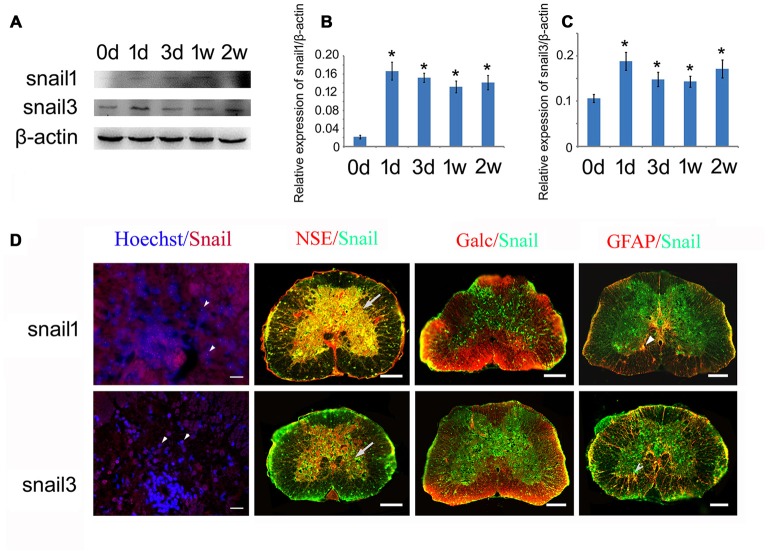
**Expression analysis of Snail1 and Snail3 proteins in the gecko spinal cord. (A)** Western blot of Snail1/3 in the spinal cord following tail amputation at 0 day, 1 day, 3 days, 1 week and 2 weeks, repectively; **(B,C)** are statistic analysis of **(A)**; **(D)** immunohistochemistry showing colocalization of Snail1 and Snail3 with Hoechst, neuron-specific enolase (NSE)- (arrows), Galc- and GFAP-positive cells (arrowheads). Data are expressed as mean ± SEM; *p < 0.05. Scale bars, 20 μm in Hoechst/Snail staining; 50 μm in others.

We first examined the expression of Snail paralogs in different gecko tissues by RT-PCR. Results revealed that *Snail1*, *Snail2* and *Snail3* were ubiquitously expressed in the brain, spinal cord, heart, liver, testis and ovary (Figures 2A–C). To understand the potential roles of Snail paralogs in the spontaneously regenerating spinal cord, gecko tail was detached at the sixth caudal vertebra, and 0.5 cm segments at the injured sites were collected at 0 day, 1 day, 3 days, 1 week and 2 weeks, respectively. Expression analysis of Snail paralogs displayed that both *Snail1* and *Snail3* were markedly upregulated in the cord from 1 day onwards after lesion, while *Snail2* decreased at 3 days, 1 week and 2 weeks, (Figures 2D–F). We therefore focused our attention on the roles of Snail1 and Snail3 in the regenerating spinal cord, while ignoring those of Snail2 for its dispensable action in embryonic, especially in neural crest development (Jiang et al., 1998). The expression level of Snail1/3 protein showed a consistency with those of the transcription, except at 2 weeks due to differential translation mechanisms (Figures 3A–C). Subsequent immunostaining in the sections of the same segments demonstrated that both Snail1 and Snail3 located in the nucleus, and colocalized with NSE- and astrocyte-specific GFAP-positive cells, but not with oligodendrocyte-specific galactocerebroside-positive cells (Figure 3D). The staining intensity in the two cell types is also enhanced after SCI (data not shown), indicating that both neurons and astrocytes are potentially regulated by Snail1 and Snail3 during the spinal cord regeneration.

### Enforced Expression of Snail1/3 Rescues Neuronal Apoptosis Induced by Glucose Deprivation

**Figure 4:**
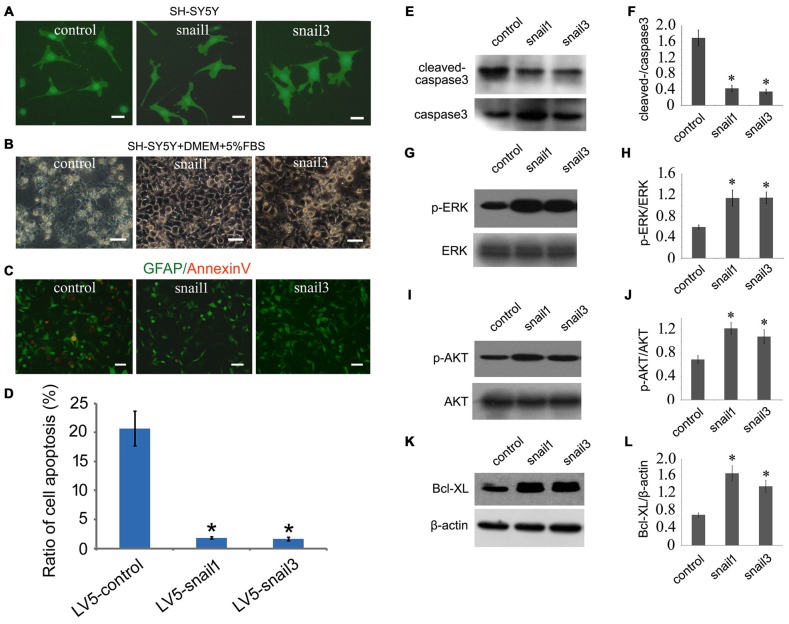
**Snail1 and Snail3 protect SH-SY5Y cells from apoptosis induced by glucose deprivation (GD). (A)** Showing morphology of Snail1- and Snail3-expressing SH-SY5Y cells. Note that GFP and Snail are not fusion protein in LV5 vector; **(B)** morphology of Snail1- and Snail3-expressing SH-SY5Y cells under stress of GD for 24 h; **(C)** immunostaining of annexin-V; **(D)** statistic analysis of **(C)** in 20 visual fields; **(E–L)** Western blot analysis of cleaved-caspase3 **(E,F)**, p-ERK **(G,H)**, p-Akt **(I,J)** and Bcl-xL **(K,L)** in SH-SY5Y cells cultured by GD for 24 h. Data are expressed as mean ± SEM; *p < 0.01. Scale bars, 25 μm in **(A)**, 50 μm in **(B,C)**.

To unveil the physiological roles of increased expression of Snail1 and Snail3 in neurons following spinal cord transection, we overexpressed Snail1 and Snail3 in the differentiated SH-SY5Y cells by lentivirus. Compared with the controls, the length of neurite or cell morphology has not been changed remarkably (Figure 4A). Given that a large amount of neurons survived from axonal injury in gecko spinal cord, we turned to account for its function on anti-apoptosis, which has been mentioned in the hematopoietic progenitor cells and radial glial precursor cells (Wu et al., 2005; Zander et al., 2014). GD is a convictive model for inducing cell apoptosis *in vitro* (Ferretti et al., 2016). Following glucose free for 24 h in the culture medium, the control cells suffered markedly apoptosis, as evidenced by morphology and Annexin-V staining (Figures 4B–D). Whereas cells transfected with Snail1 or Snail3 lentivirus showed an increased ratio of survival, indicating the important anti-apoptotic roles of both proteins on injured neurons (Figures 4B–D).

We next sought to investigate the mechanism of Snail-expressing cells resistant to GD. Biochemical results showed that the activity of cleaved caspase3 was significantly decreased, and both the MAPK (p-ERK) and PI3K (p-AKT) pathways were highly active in Snail1- and snail3-expressing cells (Figures 4E–J). It has been well known that MEK/Erk and PI3-K/Akt pathways can mediate the upregulation of Bcl-xL, a death-inhibitory member of the Bcl-2 family that blocks the stress-induced release of cytochrome c (Ramljak et al., 2003; Vega et al., 2004). As such, we determined the expression of Bcl-xL in the Snail1- and Snail3-expressing SH-SY5Y cells. As expected, it was significantly upregulated in comparison with those of the control (Figures 4K,L). The data indicate that activation of the MEK/Erk and PI3-K/Akt ascribed to enforced Snail expression, contributes to the survival properties following GD.

### Overexpression of Snail1/3 Promotes Migration of Astrocytes

**Figure 5:**
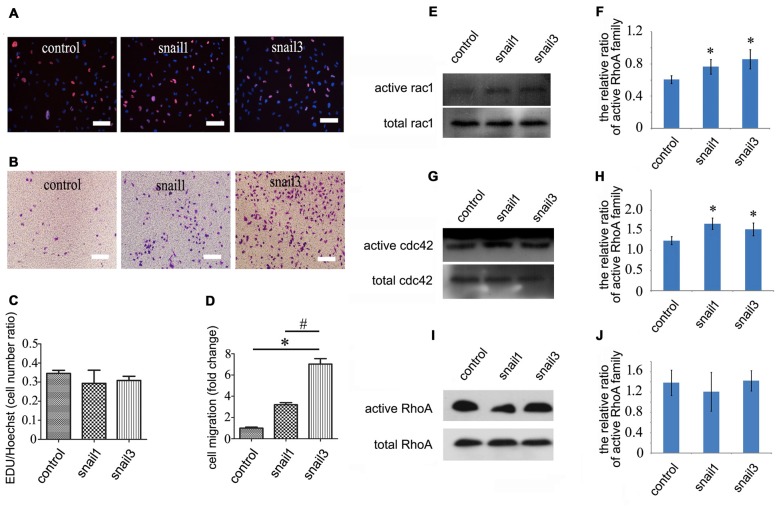
**Effects of Snail1 and Snail3 on proliferation and migration of astrocytes *in vitro*. (A)** Astrocytes were transfected with Snail1 and Snail3 for 24 h, and were detected by EDU incorporation for proliferation; **(B)** migration determination of Snail1- and Snail3-expressing astrocytes at 30 h by Transwell; **(C)** statistic analysis of **(A)**; **(D)** statistic analysis of **(B)**;** (E–J)** determination of Rac1/Cdc42/RhoA in signaling activation of Snail1- and Snail3-expressing astrocytes at 24 h. **(F)**, **(H,J)** are statistic analysis of **(E)**, **(G,I)**, respectively. Data are expressed as mean ± SEM; *p < 0.01; ^#^*P* < 0.01. Scale bars, 10 μm in **(A,B)**.

Both Snail1 and Snail3 expression were also upregulated in astrocytes following gecko spinal cord amputation. Accordingly, we examined their effects on gecko Gsn1 cell line *in vitro* following lentivirus overexpression. Snail1- and Snail3-expressing astrocytes have not shown a significant increase in proliferation, as assayed by EDU incorporation (Figures 5A,C). However, transwell experiments demonstrated that strengthening expression of Snail1 and Snail3 facilitated the migration of astrocytes (Figures 5B,D).

Cell migration and morphological changes are implicated in the actin cytoskeleton remodeling, and this process is controlled by RhoA/Cdc42/Rac1 pathways (Pawlak and Helfman, 2001). To determine whether Snail affects the status of RhoA/Cdc42/Rac1 in astrocytes, GTP-bound RhoA/Cdc42/Rac1 was investigated by pull-down assay. The level of active Cdc42 and Rac1 was found to be increased markedly in Snail1- and Snail3-expressing cells (Figures 5E–H), whereas the active GTP-RhoA remained unaltered (Figures 5I,J). The data indicate that Snail1 and Snail3 mediate astrocytic migration through Cdc42/Rac1 signaling.

### Snail1/3 Upregulates EMT-Related Markers in the Astrocytes

**Figure 6:**
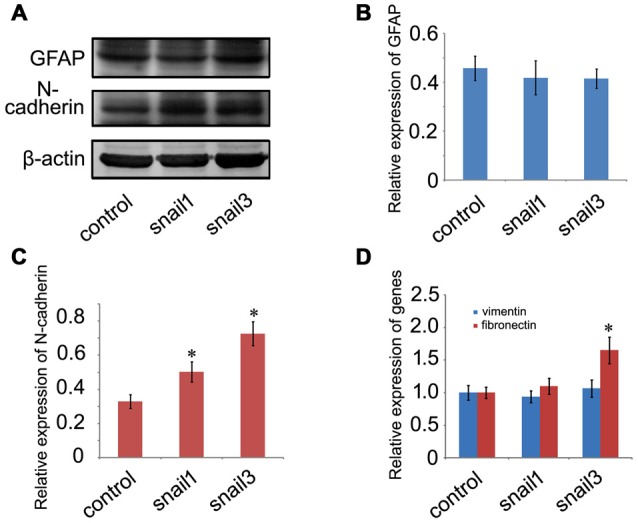
**Determination of epithelial-mesenchymal transition (EMT)-related markers in Snail1- and Snail3-expressing astrocytes at 24 h. (A)** Showing unchanged expression of GFAP and decreased expression of N-cadherin; **(B,C)** statistic analysis of **(A)**; **(D)** expression analysis of *vimentin* and *fibronectin* by RT-PCR. Data are expressed as mean ± SEM; *p < 0.01.

The most prominent function for the Snail is its involvement in inducing EMT during the development and tumor progression, where it down-regulates epithelial genes and up-regulates mesenchymal genes (Zeisberg and Neilson, 2009). It has been shown that Scratch1 and Scratch2 regulate neuronal migration onset *via* an EMT-like mechanism (Itoh et al., 2013), whereas EMT-related markers in glia lineages are still unknown. Astrocytes are derived from heterogeneous populations of progenitor cells in the neuroepithelium of the developing CNS (Rowitch and Kriegstein, 2010), hinting at the same ectodermal origin with epithelial cells. Therefore, we began to understand whether Snail participated in the regulation of EMT-related markers in astrocytes. Both Snail1 and Snail3 have not facilitated the expression of GFAP, a marker of reactive astrocytes in the mammalian CNS (Figures 6A,B), but they enhanced the expression of N-cadherin (Figures 6A,C), a similar pattern emerging in the EMT process (Zeisberg and Neilson, 2009). Neither Snail1 nor Snail3 was able to affect the expression level of *vimentin*, but Snail3 additionally stimulated the transcription of *fibronectin* (Figure 6D). The data indicate that Snail1 and Snail3 are capable of activation of several EMT-related markers by avoiding reactive gliosis.

### TGFβ1 Activates Snail Expression during the Gecko Spinal Cord Regeneration

**Figure 7:**
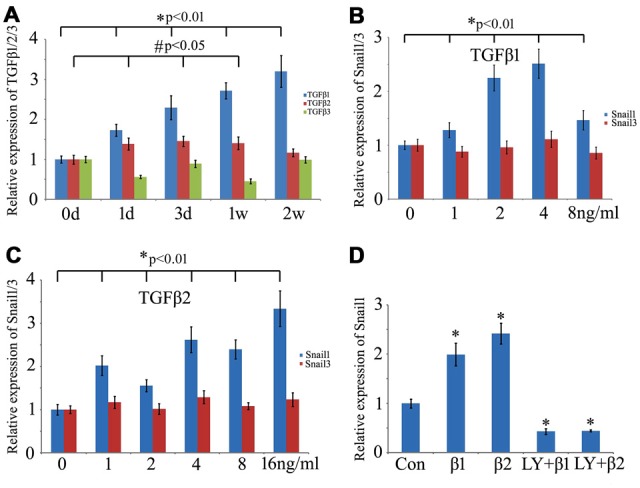
**Effects of transforming growth factor-beta (TGFβ) on expression of Snail1 and Snail3 in the Gsn1 cells line. (A)** RT-PCR analysis of TGFβ1, TGFβ2 and TGFβ3 expression at 0 day, 1 day, 3 days, 1 week and 2 weeks, respectively, following Spinal cord injury (SCI); **(B)** effects of different concentration of recombinant TGFβ1 protein on expression of Snail1 and Snail3; **(C)** effects of different concentration of recombinant TGFβ2 protein on expression of Snail1 and Snail3; **(D)** TGFβ receptor inhibitor LY2109761 blocked the action of TGFβ1 and TGFβ2 on Snail1. Data are expressed as mean ± SEM; *p < 0.01; ^#^*P* < 0.05.

TGFβ, a pleiotrophic cytokine, has been shown to mediate Snail expression by binding to the constitutively active type II serine/threonine kinase receptor (TβRII), whereby it heteromerizes with and activates the type I serine/threonine kinase TGFβ receptor (TβRI; Barrallo-Gimeno and Nieto, 2005; Thakur et al., 2014). To ascertain specific TGFβ types involved in the regulation of snail expression in the regenerating spinal cord, we first examined the expression changes of *TGFβ1*, *TGFβ2* and *TGFβ3* in response to the cord injury. Both *TGFβ1* and *TGFβ2* showed an increase in the regenerating spinal cord, which might correlate with the transcriptional activation of Snail1/3 (Figure 7A). Cultured Gsn1 cells were further treated with 0, 1, 2, 4, 8 ng/ml and 16 ng/ml recombinant TGF*β*1 or TGF*β*2 proteins for 2 h, respectively. Transcriptional analysis revealed that *Snail1* was activated by TGF*β*1 or TGF*β*2 in a concentration-dependent manner (Figures 7B,C). Addition of 10 μmol/L TGF*β* receptor inhibitor LY2109761 efficiently blocked these actions (Figure 7D). In contrast, expression of *Snail3* remains unchanged following treatment with recombinant TGFβ1 or TGF*β*2 protein, suggesting additional factor(s) responsible for its activation.

## Discussion

Epimorphic regeneration in regenerative animals attracts great attention due to their high-fidelity performance in repairing the lost structures (Brockes and Kumar, 2008).* Gekko japonicus* has a remarkable ability to regenerate amputated tail including major axial structures such as spinal cord, cartilage, muscles and spinal nerves, etc. (Wang et al., 2012; Zhou et al., 2013; Bai et al., 2015; Liu et al., 2015). The animal is becoming a new experimental model in the investigation of spinal cord regeneration (Szarek et al., 2016). The regenerating spinal cord (ependymal tube) begins to penetrate into the blastema at 10–15 days after tail amputation (McLean and Vickaryous, 2011; Delorme et al., 2012). Concomitant with this process, massive either newly generated or surviving neurons grow axons to innervate the correct targets. A transient population of GFAP-positive cells is observed in the newly formed apical ampulla, however, they do not develop a glial scar which usually emerges in mammals (Alibardi, 2014). In the present study, we have shown that Snail1 and Snail3 are implicated in neuronal anti-apoptosis and astrocytic migration in the *in vitro* model, suggesting their beneficial functions in repairing CNS.

Survival of injured neurons is an indispensible strategy for successful spinal cord regeneration, along with neurogenesis (Lee-Liu et al., 2013; Zhou et al., 2013). Snail offers protection from both stress-induced cell death and that evoked by pro-apoptotic signals. Works in *C. elegans* revealed a developmental role for Snail in cell survival, by regulation of asymmetric precursor division (Hatzold and Conradt, 2008). During the embryonic development of chicken, overexpression of Snail protects the neural crest from the naturally occurring cell death (Vega et al., 2004). In the developing murine cortex, Snail promotes cell survival by antagonizing a p53-dependent death pathway (Zander et al., 2014). Snail is also found to be a critical switch that prevents apoptosis of hematopoietic progenitors by trans-activation of *puma* (Wu et al., 2005). Thus, the roles of Snail family members in conferring resistance to cell death are likely to be conserved in the development of different species, and even extending to the spinal cord regeneration.

It is interesting to note that the glial scar formation is absent from regenerative model organisms, which has been considered as the main extrinsic impediment for axonal regrowth and functional recovery after SCI in mammals (Lee-Liu et al., 2013). Anamniotes are thought to be devoid of the so-called stellate astrocytes. Instead, the radial glia subserves many of the functions of differentiated astrocytes (Lyons and Talbot, 2014). Our works on gecko astrocytes have shown that these glial cells exhibit distinct characteristics to the mammal counterparts, such as unaltered GFAP expression, absent from glial scar formation and differential transcriptional profiles in response to mechanical stimuli, suggesting a potential plasticity during the spinal cord regeneration (Gao et al., 2010; Gu et al., 2015). In fish, CNS injuries promote glial proliferation and migration into the lesion site, where they facilitate regeneration by providing a bridging substrate for growing axons (Goldshmit et al., 2012). Works in newts indicate that the meningeal fibroblasts and glial cells migrate into the injury site along with endothelial cells and create a substrate on which the axons can regrow (Zukor et al., 2011). Whether Snail1/3-mediated astrocytes exert similar functions during the gecko spinal cord regeneration deserves further investigation.

Three members of the Snail family have displayed differential functions during embryonic development and pathophysiological process. In mouse embryos, Snail1 is essential for gastrulation, while Snail2 is dispensable for embryonic development (Jiang et al., 1998; Carver et al., 2001). A large number of studies have demonstrated that Snail1 and Snail2 are expressed in a variety of tumors, with distinct roles in tumor progression and metastasis (Olmeda et al., 2007, 2008). The reasons are partly ascribed to the differential role of Snail1 and Snail2 Zinc Fingers in E-cadherin repression and EMT (Villarejo et al., 2014). Snail3 is specifically detected in skeletal muscle and thymus at a relatively late stage of mouse development, and presents a redundant function with Snail2 in regards to B and T cell differentiation (Zhuge et al., 2005). So, it cannot be excluded that these Snail members may play differential roles in spontaneous spinal cord regeneration. In the present study, it has been shown that expression of *Snail2* is downregulated after SCI, and the potential functions are still elusive. Inhibitor of Snail2 might be a potential target for improving spinal cord regeneration.

TGFβ family members are actively involved in control of cellular proliferation, apoptosis, cell migration and adhesion. Among which, TGFβ1 induces Snail1 in hepatocytes, in the palate, and in epithelial and mesothelial cells, while TGFβ2 induces Snail1 in the developing mouse skin and Snail2 during heart development (Barrallo-Gimeno and Nieto, 2005). TGFβ3 null mutant mice show a cleft palate phenotype, due to upregulation of TGFβ1 expression in the mesenchyme. Through that, it induces the expression of Snail genes promoting the survival of the medial edge epithelial cells and permitting their subsequent differentiation into keratinized stratified epithelium (Martínez-Alvarez et al., 2004). Collectively, the TGFβ superfamily is able to induce the expression of Snail1 and Snail2 in a tissue-specific manner. To date, little is known about the Snail3 regulation by this family member. TGFβ/activin signaling has been proved to be active following gecko tail amputation with a potential role in inducing EMT during multi-tissue regeneration (Gilbert et al., 2013). We also showed that both TGFβ1 and TGFβ2 were upregulated in response to injury, which in turn induced expression of Snail1, rather than Snail3. The results suggest that cytokines not limited to TGFβ, may participate in the protecting neurons from apoptosis and promoting the migration of glial cells *via* Snail signaling.

**Figure 8:**
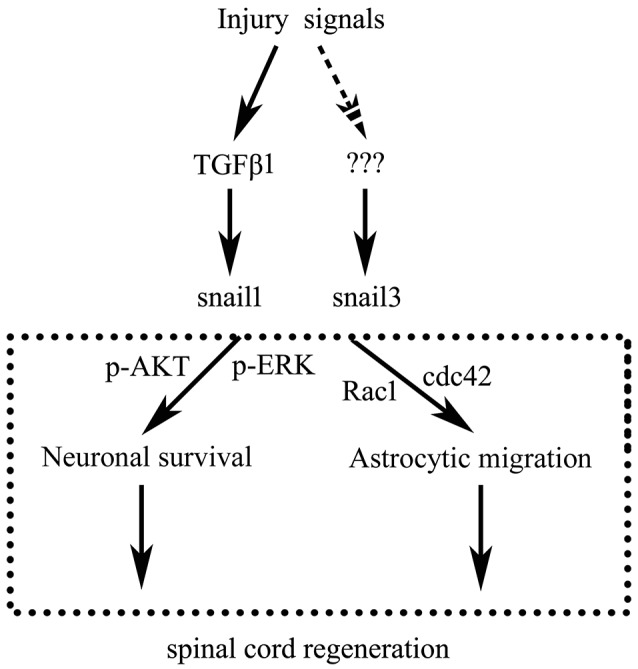
**Illustration of Snail1 and Snail3 function on spontaneous spinal cord regeneration**.

In conclusion, as illustrated in Figure 8, SCI activates both Snail1 and Snail3 through cytokines including TGFβ, which in turn act roles of apoptotic repression in neurons and migratory enhancement in glial cells, thus might promote spontaneous spinal cord regeneration.

## Author Contributions

YjunW designed this work and wrote the article. TS and YjunW performed the experiments. TS, YjunW, YjieW, QZ, XB, SW, XZ, WW, YY, YL and ML analyzed the data. XG joined discussions. All authors have approved the present version of the manuscript and have agreed to be accountable for all aspects of the work regarding questions related to the accuracy or integrity of any part of the work.

## Conflict of Interest Statement

The authors declare that the research was conducted in the absence of any commercial or financial relationships that could be construed as a potential conflict of interest.

## References

[B1] Alibardi L. (2014). Histochemical, biochemical and cell biological aspects of tail regeneration in lizard, an amniote model for studies on tissue regeneration. Prog. Histochem. Cytochem. 48, 143–244. 10.1016/j.proghi.2013.12.001 24387878

[B2] Alibardi L. (2016). Immunolocalization of FGF8/10 in the apical epidermal peg and blastema of the regenerating tail in lizard marks this apical growing area. Ann. Anat. 206, 14–20. 10.1016/j.aanat.2016.03.010 27113329

[B3] Alibardi L. (2017). Wnt-1 immunodetection in the regenerating tail of lizard suggests it is involved in the proliferation and distal growth of the blastema. Acta Histochem. [Epub ahead of print]. 10.1016/j.acthis.2017.01.001 28233575

[B4] Altschul S. F. Madden T. L. Schäffer A. A. Zhang J. Zhang Z. Miller W.. (1997). Gapped BLAST and PSI-BLAST: a new generation of protein database search programs. Nucleic Acids Res. 25, 3389–3402. 10.1093/nar/25.17.3389 9254694 PMC146917

[B5] Alunni A. Vaccari S. Torcia S. Meomartini M. E. Nicotra A. Alfei L. (2005). Characterization of glial fibrillary acidic protein and astroglial architecture in the brain of a continuously growing fish, the rainbow trout. Eur. J. Histochem. 49, 157–166. 10.4081/940 15967744

[B6] Bai X. Wang Y. Man L. Zhang Q. Sun C. Hu W.. (2015). CD59 mediates cartilage patterning during spontaneous tail regeneration. Sci. Rep. 5:12798. 10.1038/srep12798 26238652 PMC4523838

[B7] Barrallo-Gimeno A. Nieto M. A. (2005). The Snail genes as inducers of cell movement and survival: implications in development and cancer. Development 132, 3151–3161. 10.1242/dev.01907 15983400

[B8] Beck C. W. Christen B. Slack J. M. (2003). Molecular pathways needed for regeneration of spinal cord and muscle in a vertebrate. Dev. Cell 5, 429–439. 10.1016/s1534-5807(03)00233-8 12967562

[B9] Brockes J. P. Kumar A. (2008). Comparative aspects of animal regeneration. Annu. Rev. Cell Dev. Biol. 24, 525–549. 10.1146/annurev.cellbio.24.110707.175336 18598212

[B10] Burland T. G. (2000). DNASTAR’S Lasergene sequence analysis software. Methods Mol. Biol. 132, 71–91. 10.1385/1-59259-192-2:71 10547832

[B11] Carver E. A. Jiang R. Lan Y. Oram K. F. Gridley T. (2001). The mouse snail gene encodes a key regulator of the epithelial-mesenchymal transition. Mol. Cell. Biol. 21, 8184–8188. 10.1128/mcb.21.23.8184-8188.2001 11689706 PMC99982

[B12] Delorme S. L. Lungu I. M. Vickaryous M. K. (2012). Scar-free wound healing and regeneration following tail loss in the leopard gecko, *Eublepharis macularius*. Anat. Rec. (Hoboken) 295, 1575–1595. 10.1002/ar.22490 22933425

[B13] Díaz-Quiroz J. F. Echeverri K. (2013). Spinal cord regeneration: where fish, frogs and salamanders lead the way, can we follow? Biochem. J. 451, 353–364. 10.1042/BJ20121807 23581406

[B14] Dong Y. Gu Y. Huan Y. Wang Y. Liu Y. Liu M.. (2013). HMGB1 protein does not mediate the inflammatory response in spontaneous spinal cord regeneration: a hint for CNS regeneration. J. Biol. Chem. 288, 18204–18218. 10.1074/jbc.m113.463810 23649623 PMC3689963

[B15] Egar M. Singer M. (1972). The role of ependyma in spinal cord regeneration in the urodele, *Triturus*. Exp. Neurol. 37, 422–430. 10.1016/0014-4886(72)90085-4 4637959

[B16] Ferretti A. C. Tonucci F. M. Hidalgo F. Almada E. Larocca M. C. Favre C. (2016). AMPK and PKA interaction in the regulation of survival of liver cancer cells subjected to glucose starvation. Oncotarget 7, 17815–17828. 10.18632/oncotarget.7404 26894973 PMC4951252

[B17] Ferretti P. Zhang F. O’Neill P. (2003). Changes in spinal cord regenerative ability through phylogenesis and development: lessons to be learnt. Dev. Dyn. 226, 245–256. 10.1002/dvdy.10226 12557203

[B18] Gao D. Wang Y. Liu Y. Ding F. Gu X. Li Z. (2010). The molecular cloning of glial fibrillary acidic protein in Gekko japonicus and its expression changes after spinal cord transection. Cell. Mol. Biol. Lett. 15, 582–599. 10.2478/s11658-010-0029-x 20711818 PMC6275668

[B19] Gilbert R. W. Vickaryous M. K. Viloria-Petit A. M. (2013). Characterization of TGFβ signaling during tail regeneration in the leopard Gecko (*Eublepharis macularius*). Dev. Dyn. 242, 886–896. 10.1002/dvdy.23977 23592270

[B20] Goldshmit Y. Sztal T. E. Jusuf P. R. Hall T. E. Nguyen-Chi M. Currie P. D. (2012). Fgfdependent glial cell bridges facilitate spinal cord regeneration in zebrafish. J. Neurosci. 32, 7477–7492. 10.1523/JNEUROSCI.0758-12.2012 22649227 PMC6703582

[B21] Grimes H. L. Chan T. O. Zweidler-McKay P. A. Tong B. Tsichlis P. N. (1996). The Gfi-1 proto-oncoprotein contains a novel transcriptional repressor domain, SNAG, and inhibits G1 arrest induced by interleukin-2 withdrawal. Mol. Cell. Biol. 16, 6263–6272. 10.1128/mcb.16.11.6263 8887656 PMC231629

[B22] Gu Y. Yang J. Chen H. Li J. Xu M. Hua J.. (2015). Different astrocytic activation between adult gekko japonicus and rats during wound healing *in vitro*. PLoS One 10:e0127663. 10.1371/journal.pone.0127663 26020931 PMC4447339

[B23] Gupta P. B. Kuperwasser C. Brunet J. P. Ramaswamy S. Kuo W. L. Gray J. W.. (2005). The melanocyte differentiation program predisposes to metastasis after neoplastic transformation. Nat. Genet. 37, 1047–1054. 10.1038/ng1634 16142232 PMC1694635

[B24] Hatzold J. Conradt B. (2008). Control of apoptosis by asymmetric cell division. PLoS Biol. 6:e84. 10.1371/journal.pbio.0060084 18399720 PMC2288629

[B25] Hemavathy K. Ashraf S. I. Ip Y. T. (2000). Snail/slug family of repressors: slowly going into the fast lane of development and cancer. Gene 257, 1–12. 10.1016/s0378-1119(00)00371-1 11054563

[B26] Inoue A. Seidel M. G. Wu W. Kamizono S. Ferrando A. A. Bronson R. T.. (2002). Slug, a highly conserved zinc finger transcriptional repressor, protects hematopoietic progenitor cells from radiation-induced apoptosis *in vivo*. Cancer Cell 2, 279–288. 10.1016/s1535-6108(02)00155-1 12398892

[B27] Itoh Y. Moriyama Y. Hasegawa T. Endo T. A. Toyoda T. Gotoh Y. (2013). Scratch regulates neuronal migration onset via an epithelial-mesenchymal transition-like mechanism. Nat. Neurosci. 16, 416–425. 10.1038/nn.3336 23434913

[B28] Jiang R. Lan Y. Norton C. R. Sundberg J. P. Gridley T. (1998). The Slug gene is not essential for mesoderm or neural crest development in mice. Dev. Biol. 198, 277–285. 10.1006/dbio.1998.8909 9659933

[B29] Kawai H. Arata N. Nakayasu H. (2001). Three-dimensional distribution of astrocytes in zebrafish spinal cord. Glia 36, 406–413. 10.1002/glia.1126 11746776

[B30] Kerner P. Hung J. Béhague J. Le Gouar M. Balavoine G. Vervoort M. (2009). Insights into the evolution of the snail superfamily from metazoan wide molecular phylogenies and expression data in annelids. BMC Evol. Biol. 9:94. 10.1186/1471-2148-9-94 19426549 PMC2688512

[B31] Kurrey N. K. K A. Bapat S. A. (2005). Snail and Slug are major determinants of ovarian cancer invasiveness at the transcription level. Gynecol. Oncol. 97, 155–165. 10.1016/j.ygyno.2004.12.043 15790452

[B32] Le Douarin N. M. Dupin E. Ziller C. (1994). Genetic and epigenetic control in neural crest development. Curr. Opin. Genet. Dev. 4, 685–695. 10.1016/0959-437x(94)90135-p 7849508

[B33] Lee-Liu D. Edwards-Faret G. Tapia V. S. Larraín J. (2013). Spinal cord regeneration: lessons for mammals from non-mammalian vertebrates. Genesis 51, 529–544. 10.1002/dvg.22406 23760835

[B34] Liu Y. Zhou Q. Wang Y. Luo L. Yang J. Yang L.. (2015). *Gekko japonicus* genome reveals evolution of adhesive toe pads and tail regeneration. Nat. Commun. 6:10033. 10.1038/ncomms10033 26598231 PMC4673495

[B35] Lozito T. P. Tuan R. S. (2015). Lizard tail regeneration: regulation of two distinct cartilage regions by Indian hedgehog. Dev. Biol. 399, 249–262. 10.1016/j.ydbio.2014.12.036 25596336

[B36] Lozito T. P. Tuan R. S. (2016). Lizard tail skeletal regeneration combines aspects of fracture healing and blastema-based regeneration. Development 143, 2946–2957. 10.1242/dev.129585 27387871 PMC5004880

[B37] Lyons D. A. Talbot W. S. (2014). Glial cell development and function in zebrafish. Cold Spring Harb. Perspect. Biol. 7:a020586. 10.1101/cshperspect.a020586 25395296 PMC4315925

[B38] Manzanares M. Locascio A. Nieto M. A. (2001). The increasing complexity of the Snail gene superfamily in metazoan evolution. Trends Genet. 17, 178–181. 10.1016/s0168-9525(01)02232-6 11275308

[B39] Martínez-Alvarez C. Blanco M. J. Pérez R. Rabadán M. A. Aparicio M. Resel E.. (2004). Snail family members and cell survival in physiological and pathological cleft palates. Dev. Biol. 265, 207–218. 10.1016/j.ydbio.2003.09.022 14697364

[B40] Mayor R. Morgan R. Sargent M. G. (1995). Induction of the prospective neural crest of *Xenopus*. Development 121, 767–777. 7720581 10.1242/dev.121.3.767

[B41] McLean K. E. Vickaryous M. K. (2011). A novel amniote model of epimorphic regeneration: the leopard gecko, *Eublepharis macularius*. BMC Dev. Biol. 11:50. 10.1186/1471-213x-11-50 21846350 PMC3180301

[B42] Nieto M. A. (2002). The snail superfamily of zinc-finger transcription factors. Nat. Rev. Mol. Cell Biol. 3, 155–166. 10.1038/nrm757 11994736

[B43] Nieto M. A. Sargent M. G. Wilkinson D. G. Cooke J. (1994). Control of cell behavior during vertebrate development by Slug, a zinc finger gene. Science 264, 835–839. 10.1126/science.7513443 7513443

[B44] Olmeda D. Montes A. Moreno-Bueno G. Flores J. M. Portillo F. Cano A. (2008). Snai1 and Snai2 collaborate on tumor growth and metastasis properties of mouse skin carcinoma cell lines. Oncogene 27, 4690–4701. 10.1038/onc.2008.118 18408755

[B45] Olmeda D. Moreno-Bueno G. Flores J. M. Fabra A. Portillo F. Cano A. (2007). Snail is required for tumor growth and lymph node metastasis of human breast carcinoma. Cancer Res. 67, 11721–11731. 10.1158/0008-5472.can-07-2318 18089802

[B46] Pawlak G. Helfman D. M. (2001). Cytoskeletal changes in cell transformation and tumorigenesis. Curr. Opin. Genet. Dev. 11, 41–47. 10.1016/s0959-437x(00)00154-4 11163149

[B47] Pérez-Mancera P. A. González-Herrero I. Pérez-Caro M. Gutiérrez-Cianca N. Flores T. Gutiérrez-Adán A.. (2005). *SLUG* in cancer development. Oncogene 24, 3073–3082. 10.1038/sj.onc.1208505 15735690

[B48] Popovich P. G. Longbrake E. E. (2008). Can the immune system be harnessed to repair the CNS? Nat. Rev. Neurosci. 9, 481–493. 10.1038/nrn2398 18490917

[B49] Ramljak D. Coticchia C. M. Nishanian T. G. Saji M. Ringel M. D. Conzen S. D. (2003). Epidermal growth factor inhibition of c-Myc-mediated apoptosis through Akt and Erk involve Bcl-xL upregulation in mammary epithelial cells. Exp. Cell Res. 287, 397–410. 10.1016/s0014-4827(03)00135-6 12837294

[B50] Rasmussen J. P. Sagasti A. (2017). Learning to swim, again: axon regeneration in fish. Exp. Neurol. 287, 318–330. 10.1016/j.expneurol.2016.02.022 26940084

[B51] Rowitch D. H. Kriegstein A. R. (2010). Developmental genetics of vertebrate glial-cell specification. Nature 468, 214–222. 10.1038/nature09611 21068830

[B52] Sefton M. Sánchez S. Nieto M. A. (1998). Conserved and divergent roles for members of the Snail family of transcription factors in the chick and mouse embryo. Development 125, 3111–3121. 9671584 10.1242/dev.125.16.3111

[B53] Singer M. Nordlander R. H. Egar M. (1979). Axonal guidance during embryogenesis and regeneration in the spinal cord of the newt: the blueprint hypothesis of neuronal pathway patterning. J. Comp. Neurol. 185, 1–21. 10.1002/cne.901850102 429610

[B54] Stocum D. L. (1984). The urodele limb regeneration blastema. Determination and organization of the morphogenetic field. Differentiation 27, 13–28. 10.1111/j.1432-0436.1984.tb01403.x 6381198

[B55] Szarek D. Marycz K. Lis A. Zawada Z. Tabakow P. Laska J.. (2016). Lizard tail spinal cord: a new experimental model of spinal cord injury without limb paralysis. FASEB J. 30, 1391–1403. 10.1096/fj.15-272468 26667043

[B56] Thakur N. Gudey S. K. Marcusson A. Fu J. Y. Bergh A. Heldin C. H.. (2014). TGFβ-induced invasion of prostate cancer cells is promoted by c-Jun-dependent transcriptional activation of Snail1. Cell Cycle 13, 2400–2414. 10.4161/cc.29339 25483191 PMC4128885

[B57] Vega S. Morales A. V. Ocaña O. H. Valdés F. Fabregat I. Nieto M. A. (2004). Snail blocks the cell cycle and confers resistance to cell death. Genes Dev. 18, 1131–1143. 10.1101/gad.294104 15155580 PMC415638

[B58] Villarejo A. Cortés-Cabrera A. Molina-Ortíz P. Portillo F. Cano A. (2014). Differential role of Snail1 and Snail2 zinc fingers in E-cadherin repression and epithelial to mesenchymal transition. J. Biol. Chem. 289, 930–941. 10.1074/jbc.M113.528026 24297167 PMC3887216

[B59] Wang Y. Dong Y. Song H. Liu Y. Liu M. Yuan Y.. (2012). Involvement of gecko SNAP25b in spinal cord regeneration by promoting outgrowth and elongation of neurites. Int. J. Biochem. Cell Biol. 44, 2288–2298. 10.1016/j.biocel.2012.09.011 23010346

[B60] Wu W. S. Heinrichs S. Xu D. Garrison S. P. Zambetti G. P. Adams J. M.. (2005). Slug antagonizes p53-mediated apoptosis of hematopoietic progenitors by repressing puma. Cell 123, 641–653. 10.1016/j.cell.2005.09.029 16286009

[B61] Zander M. A. Burns S. E. Yang G. Kaplan D. R. Miller F. D. (2014). Snail coordinately regulates downstream pathways to control multiple aspects of mammalian neural precursor development. J. Neurosci. 34, 5164–5175. 10.1523/JNEUROSCI.0370-14.2014 24719096 PMC3983799

[B62] Zeisberg M. Neilson E. G. (2009). Biomarkers for epithelial-mesenchymal transitions. J. Clin. Invest. 119, 1429–1437. 10.1172/JCI36183 19487819 PMC2689132

[B63] Zhou Y. Xu Q. Li D. Zhao L. Wang Y. Liu M.. (2013). Early neurogenesis during caudal spinal cord regeneration in adult *Gekko japonicus*. J. Mol. Histol. 44, 291–297. 10.1007/s10735-012-9466-3 23143000

[B64] Zhuge X. Kataoka H. Tanaka M. Murayama T. Kawamoto T. Sano H.. (2005). Expression of the novel Snai-related zinc-finger transcription factor gene Smuc during mouse development. Int. J. Mol. Med. 15, 945–948. 10.3892/ijmm.15.6.945 15870897

[B65] Zukor K. A. Kent D. T. Odelberg S. J. (2011). Meningeal cells and glia establish a permissive environment for axon regeneration after spinal cord injury in newts. Neural Dev. 6:1. 10.1186/1749-8104-6-1 21205291 PMC3025934

